# Sympathetic Insanity Proceeding from the Rectum

**Published:** 1879-04

**Authors:** Ben James Baldwin


### SYMPATHETIC INSANITY PROCEEDING FROM THE RECTUM.

By BEN JAMES BALDWIN, M.D.

Between bodily conditions and mental functions there exists the closest relation, and modern psychologists have proven beyond cavil that the brain may be deranged by reason of a morbid cause of irritation in some other part of the body. Numerous instructive examples might be quoted to illustrate the manner of pathological action, and explain this intimate organic sympathy. But the influence that abdominal affections exert over the organ of mind is well known, and frequently seen in the ordinary derangement, constipation. Habitual constipation is one of the most prevalent and troublesome of functional disorders. In the female it is the bane of existence, and its sympathetic effects extend to every organ in the body, especially to the supreme nerve centre. The ordinary effects of constipation, as giddiness, gloominess of thought, headache, drowsiness, etc., are constantly met with in persons with torpid bowels, who suffer no serious results; but its sympathetic influence not unfrequently induces a greater derangement, and may terminate in permanent perversion of intellect, or even in more distressing mental alienation. The case I am to cite affords a remarkable illustration of evil consequences that may ensue from neglect of this seemingly trifling ailment, and convinces me that in mental derangements the condition of the bowels should be scrupulously looked into.

Mr. W. H. was an intelligent gentleman, of sedentary habits, who had always suffered from torpid bowels, having several times to seek medical advice. On one occasion, after an unusual lapse of time, he sought relief by some simple remedy, which did not afford the effect desired. Not being inconvenienced, he went on for several days, and then tried some similar remedy with same result. Becoming now exceedingly anxious of himself, he consulted his doctor, who, regarding him needlessly nervous, prescribed some constipation pill, which had not the slightest effect. He grew nervous, irritable and depressed; fever came on, and he took to bed. Nervous symptoms increasing, his attention was altogether withdrawn from his bowels. Forebodings of gloom oppressed his thoughts. He began to have delusions, with hallucinations of sight and hearing, and did strange things. These actions continuing, it was decided to send him to an asylum; and he was admitted into the Kings County asylum with a most acute melancholia. No one then knew about his bowels or the cause of his trouble. He soon became the subject of perpetual horror, and his delusions grew charnel. The appalling and intolerable idea of premature burial kept him in continual anguish. These ghastly dangers haunted him day and night; and his feelings were so terribly well adapted to inspire supremeness of mental distress, that it was only when the nerve centers could endure wakefulness no longer, with large doses of chloral hydrate, that we could get him asleep; for he thought that on waking he would find himself the tenant of a grave. His sleep was not peaceful, and during waking a world of phantasms rushed in upon him. The iron bars of his cell window were to him tall and gaunt figures, shrouded in the habiliments of the grave, hovering over and ready to bear him to the tomb as soon as he was again asleep. From these terrible hallucinations and illusions the patient would wildly recoil and cry aloud in the most agonizing tones of distress.

Thus he went on losing strength and rapidly being exhausted. Finally the orderly, whose duty it was to look after the habits of patients, after a stupid delay communicated the fact that this man had not had an operation since his admission. Immediate attention was directed to his bowels. Many attempts were made. At last the heroic dose of ten gtt. croton lig. with enema of ox-gall, followed by warm water enema to soften scybalæ, was sufficient. He passed a most enormous quantity of offensive fæces. This opened to him a new world. It was the dawn of psychal day. In a short while the patient recovered sufficiently to appreciate his disgusting surroundings. He rapped loudly upon his cell door. The night watch came and found him clear and intelligent. After a bath he was assigned better quarters; and it was here where we found him next morning, much to our surprise, perfectly restored. The day after patient was discharged. He was often seen in the city afterwards, and took great interest in describing his attack and his feelings while in the asylum, asserting that he was at times dully and confusedly aware of his surroundings.

Etiologically the case is very interesting. Was it due to derangement of the assimilative organs, to retention of the excrementitious products, or to cholæmic intoxication? At any rate, it might be classed as one of Du Bois Raymond's "nervous polar molecular disorders," which rapidly appear, but rapily disappear on removal of cause. *Louisville Medical News.*

